# Feasibility of oral arsenic trioxide treatment for acute promyelocytic leukemia during hemodialysis

**DOI:** 10.1007/s00277-012-1576-1

**Published:** 2012-09-30

**Authors:** Wing-Yan Au, Bonnie M. Fong, Sidney Tam, Yok-Lam Kwong

**Affiliations:** 1 Department of Medicine, Queen Mary Hospital, Professorial Block, Pokfulam Road, Hong Kong, China; 2 Department of Clinical Biochemistry, Queen Mary Hospital, Hong Kong, China

Dear Editor,

Arsenic trioxide (As_2_O_3_) is a standard medication for relapsed acute promyelocytic leukemia (APL). However, high blood arsenic levels lead to potentially fatal arrhythmias. As_2_O_3_ is renal-excreted and considered contraindicated in renal failure.

A 42-year-old man was referred for treatment of APL in first relapse, presenting with pancytopenia, impaired renal function, and septicemia. He was treated with all-trans retinoic acid (ATRA, 45 mg/m^2^/day) and oral As_2_O_3_ (10 mg/day) [1]. Progressive renal function derangement developed, necessitating reduction of oral As_2_O_3_ to 5 mg/day. On the third day, the leukocyte count increased to 8.9 × 10^9^/L, associated with bilateral pulmonary infiltrates. Features were consistent with the APL differentiation syndrome, which with the underlying septicemia led to anuric acute renal failure. ATRA was stopped. Idarubicin (6 mg/m^2^/day × 5), dexamethasone (12 mg/day × 7), and alternate daily hemodialysis were administered. Oral As_2_O_3_ at 5 mg was given after each hemodialysis and was stopped after 9 days. At 4 weeks, marrow examination confirmed complete remission. He remained anuric, and hemodialysis was continued. Oral As_2_O_3_ was re-commenced at 2 mg after each hemodialysis. Two months later, continuous ambulatory peritoneal dialysis (CAPD) was started. A maintenance regimen for APL, comprising oral As_2_O_3_ (5 mg/day) and ATRA (20 mg twice daily) [2, 3], given 2 weeks every 2 months, was administered. At 6-month follow-up, he remained in remission and was negative for the *PML-RARA* fusion gene characteristic of APL. No cardiac arrhythmias were observed at any time.

**Fig. 1:**
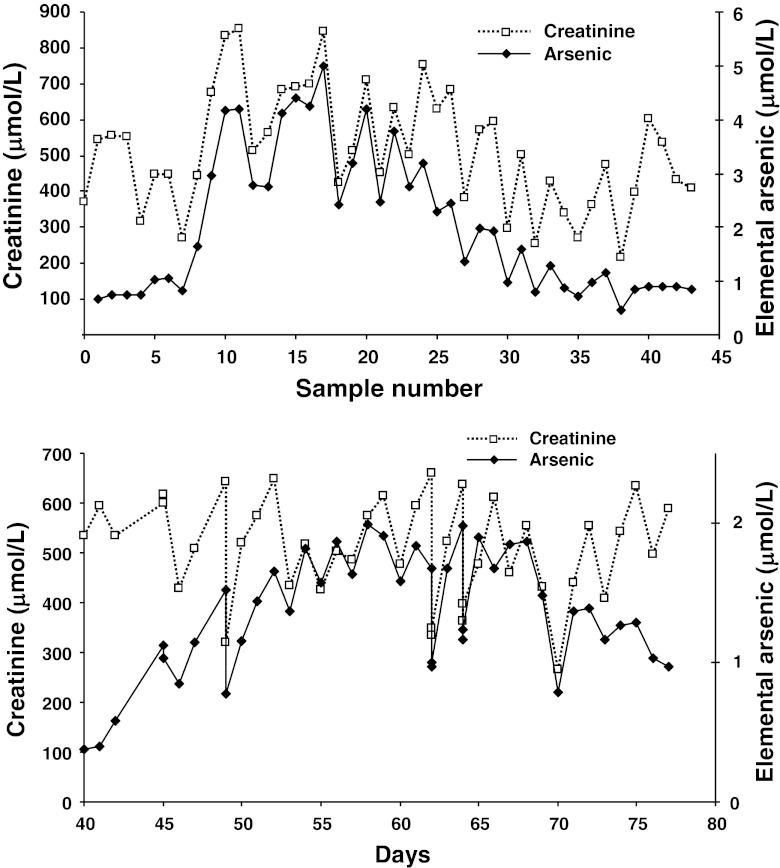
**a** Blood arsenic levels during the first 3 weeks of hemodialysis. A rapid surge of arsenic levels occurred on day 5, in parallel with a rise in creatinine and further renal function deterioration. Oral arsenic trioxide was stopped on day 9, resulting in a gradual fall of arsenic levels after hemodialysis was commenced. Days (sample number): 3 (1–4), 4 (5–7), 5 (8), 6 (9), 7 (10–13), 8 (14–16), 9 (17–19), 10 (20–21), 11 (22), 12 (23), 13 (24), 14 (24–26), 15 (28–30), 16 (31–32), 17 (33–34), 18 (35–36), 19 (37–38), 20 (39), 21 (40), 22 (41), 23 (42), and 24 (43). **b** Blood arsenic levels during the second month of hemodialysis. With the re-commencement of oral arsenic trioxide on day 42, there was a gradual increase of arsenic levels back to the therapeutic range. Arsenic levels also varied in parallel with creatinine levels during hemodialysis and had remained within the therapeutic range

Serum elemental arsenic levels were assayed by inductively coupled plasma mass spectrometry [3, 4], on blood samples sent for creatinine measurement. At 4 days, there was a sudden increase of arsenic levels, as the cellular and third-space compartments became saturated and anuria prevented arsenic excretion (Fig. 1a). The arsenic level peaked at 4,240 nmol/L, outside the therapeutic range of 500–2,000 nmol/L typical of oral As_2_O_3_. The commencement of hemodialysis and cessation of oral As_2_O_3_ resulted in a fall of arsenic levels parallel to those of creatinine. After oral As_2_O_3_ was re-commenced, arsenic levels gradually increased to the therapeutic range (Fig. 1b). As arsenic clearance in oral As_2_O_3_ therapy during CAPD had been documented previously [3], arsenic assays were not performed after peritoneal dialysis was started.

As_2_O_3_ is a key medication for patients with relapsed APL. Intravenous (i.v.) As_2_O_3_, used in most reports, may lead to lethal arrhythmias due to QT prolongation, which is directly proportional to blood arsenic levels [5]. Because i.v. As_2_O_3_ leads to a rapid surge of blood arsenic levels, arrhythmia related to QT prolongation is an important adverse effect. Oral As_2_O_3_ is slowly absorbed and results in lower blood arsenic levels. Its bioavailability, estimated by area-under-the-curve pharmacokinetically, is comparable to that of i.v. As_2_O_3_ [6]. Hence, oral As_2_O_3_ has the same efficacy, but little risk of arrhythmia [5]. In one previous report, blood arsenic levels after i.v. As_2_O_3_ were measured in an APL patient on hemodialysis [7]. The peak level was 6,450 nmol/L, a toxic level that led to termination of i.v. As_2_O_3_ therapy. It was concluded that i.v. As_2_O_3_ was contraindicated in renal failure [7]. In our case, the sudden increase in blood arsenic levels was related to an unanticipated acute renal failure. Timely cessation of oral As_2_O_3_ and institution of hemodialysis prevented further increases in arsenic levels. Subsequently, with careful dose adjustment and meticulous monitoring, oral As_2_O_3_ was safely administered during hemodialysis. The blood arsenic levels were also kept within the therapeutic range. We have also previously demonstrated the safety of oral As_2_O_3_ during CAPD. Therefore, patients on renal replacement therapy are still eligible for oral As_2_O_3_ treatment.
